# Risk of readmission in patients with schizophrenia and schizoaffective disorder newly prescribed clozapine

**DOI:** 10.1177/0269881118817387

**Published:** 2019-01-08

**Authors:** Jad Kesserwani, Giouliana Kadra, Johnny Downs, Hitesh Shetty, James H MacCabe, David Taylor, Robert Stewart, Chin-Kuo Chang, Richard D Hayes

**Affiliations:** 1 King’s College London, Institute of Psychiatry Psychology and Neuroscience, London, UK; 2 National Institute for Health Research (NIHR) Maudsley Biomedical Research Centre, London, UK; 3 South London and Maudsley National Health Service (NHS) Foundation Trust, London, UK

**Keywords:** Clozapine, readmission, atypical antipsychotics, schizophrenia, schizoaffective disorder

## Abstract

**Background:**

Insight into the effect of clozapine is limited by a lack of controlling for confounding variables in current research. Our objective was to investigate the association between clozapine prescribed at discharge, following an inpatient episode, and risk of readmission into secondary mental health services in patients with schizophrenia and schizoaffective disorder, controlling extensively for confounding variables.

**Methods:**

Clinical records from 3651 patients were analysed in a retrospective observational cohort study. Cox proportional-hazards regression models were used to assess the risk of hospital readmission. A series of sensitivity analyses were also conducted. Propensity score methods were used to address confounding-by-indication.

**Results:**

Patients on clozapine (*n*=202) had a reduced risk of readmission compared with patients on other antipsychotics (adjusted hazard ratio=0.79; 95% confidence interval: 0.64–0.99; *p*=0.043). Clozapine also had a protective effect on risk of readmission when compared with olanzapine (adjusted hazard ratio 0.76; 95% confidence interval: 0.60–0.96; *p*=0.021). The effect size remained consistent after adjusting for an array of possible confounders, as well as using propensity scores to address confounding-by-indication. A statistically significant result was also noted in all but two sensitivity analyses.

**Conclusion:**

Our findings suggest that clozapine is associated with a reduced risk of readmission into secondary mental health services.

## Introduction

Schizophrenia and schizoaffective disorder are associated with a mortality risk 2–2.5 times greater than that of the general population (Chang et al., 2010) and increased physical morbidity, which is highlighted by a 10–20-year reduction in patient life expectancy (Chang et al., 2011).

Antipsychotic medications are frequently used in the treatment of schizophrenia and schizoaffective disorder; however, despite their effectiveness in clinical practice, they have been associated with significant mortality and physical morbidity (Ray et al., 2009; Roden, 2004; Stahl et al., 2009). Mortality risk varies between antipsychotics (Crump et al., 2013), and there is emerging evidence that the choice of antipsychotic treatment may impact on a patient’s risk of premature mortality (Hayes et al., 2014). Clozapine, a second-generation antipsychotic, is effective in the treatment of treatment-resistant schizophrenia and schizoaffective disorder (Tiihonen et al., 2017; Wimberley et al., 2017). Current treatment guidelines restrict clozapine use to a third-line treatment (NICE, 2015; Remington et al., 2013) due to severe and sometimes fatal adverse effects, including agranulocytosis (Kumra et al., 2008; Nielsen et al., 2012). Despite these safety concerns, studies have reported that clozapine is associated with a decreased mortality risk, compared with other antipsychotics (Hayes et al., 2014; Wimberley et al., 2017). There are also corresponding improvements in symptoms and functioning (De Oliveira-Souza et al., 1999; Wahlbeck et al., 2009), which are the ideal measures of assessing the efficacy of clozapine, in accordance to usual clinical practice.

The heterogeneous nature of schizophrenia and schizoaffective disorder and their clinical presentations make it difficult to directly assess the effects of clozapine on symptomology in real-world settings. Given these difficulties, patterns of readmission into mental health services has been demonstrated to be a reliable marker for symptoms and functioning (Ahn et al., 2005; Conley et al., 1999; Pollack et al., 1998; Schooler et al., 1997).

Unfortunately, there is a limited pool of research focusing on the association between clozapine and risk of readmission. The majority of studies to date have found that clozapine is associated with a decreased risk of readmission (Ahn et al., 2005; Castro and Elkis, 2007; Essock et al., 1996; Gee et al., 2016; Pridan et al., 2015; Tiihonen et al., 2017; Valevski et al., 2012), although one study reported no association (Lin et al., 2006). The association with decreased risk of readmission has also been supported in a recent meta-analysis of an extensive list of studies (Land et al., 2017). However, these studies suffer from a number of limitations including neglecting to control for important confounding variables (Ahn et al., 2005; Castro and Elkis, 2007; Essock et al., 1996; Gee et al., 2016; Lin et al., 2006; Pridan et al., 2015; Valevski et al., 2012). Furthermore, confounding-by-indication is an important issue which has not been addressed adequately in a number of studies (Ahn et al., 2005; Castro and Elkis, 2007; Essock et al., 1996; Lin et al., 2006; Pridan et al., 2015). Another limitation of current research is that no studies to date have specifically investigated the effect of newly prescribed clozapine at discharge on the risk of readmission. This is relevant to clinical decision-making where clinicians have to decide whether to change or maintain medication regimens. Knowing if clozapine would reduce risk of readmission at that point in time may aid clinical decision making and lessen delays in starting patients on clozapine (Howes et al., 2012). Furthermore, most studies have only investigated schizophrenia (Ahn et al., 2005; Castro and Elkis, 2007; Lin et al., 2006; Pridan et al., 2015; Valevski et al., 2012), which does not reflect the current clinical environment, where clozapine is used in the treatment of both treatment-resistant schizophrenia and schizoaffective disorder. Also, the age ranges included in current literature are either limited (Ahn et al., 2005; Pridan et al., 2015) or not mentioned at all (Castro and Elkis, 2007; Essock et al., 1996; Lin et al., 2006). Thus, we attempted to address these limitations in this current study by using a large cohort with rich contextual information, therefore permitting to investigate and control an array of possible confounders.

### Aims of the study

The aim of this study was to investigate the effect of newly prescribed clozapine to people with schizophrenia and schizoaffective disorder on the risk of readmission into secondary mental health services, compared with those on other antipsychotics. We hypothesised that newly prescribed clozapine reduces the risk of readmission into secondary mental health services in patients with schizophrenia and schizoaffective disorder.

## Material and methods

### Setting

This study used patient data from an extensive, anonymised electronic mental health records database, the South London and Maudsley NHS Foundation Trust (SLaM) Case Register. As mental health services in the UK are provided according to geographic catchment areas under the NHS, SLaM provides mental health care for approximately 1.36 m residents from four London boroughs (Lambeth, Southwark, Lewisham and Croydon). Clinical records have been maintained electronically by SLaM services since 2006. At the time this study was conducted researchers were able to search for information on the records on over 250,000 patients using the Clinical Record Interactive Search (CRIS) system, an application drawing and anonymising clinical record data for use in research (Perera et al., 2016).

### Ethics statement

The Oxfordshire Research Ethics Committee C (08/H0606/71) approved the CRIS system as an anonymised data resource for secondary analysis, with governance provided for related projects by a patient-led oversight committee.

### Inclusion criteria

The patient cohort constituted all individuals who had been diagnosed with schizophrenia or schizoaffective disorder (World Health Organisation (WHO) International Classification of Diseases 10 (ICD-10) codes: F20, F25) in SLaM during the observation period (between 1 January 2007–31 December 2014), and who were aged between 15–95 years at index discharge (defined below). Within the observation period, these patients had at least one inpatient episode where they were discharged on an antipsychotic (either clozapine or another drug). The first discharge within the observation period where patients were prescribed an antipsychotic constituted the definition of index discharge. However, in patients prescribed clozapine, index discharge was defined as the first discharge within the observation period where they were discharged on clozapine, regardless of whether they had been discharged on another antipsychotic beforehand. Patients who were discharged on more than one antipsychotic at their index discharge were excluded from the cohort. Patients who were prescribed clozapine prior to their index discharge were also excluded since this study investigated the impact of being newly prescribed clozapine on risk of readmission.

### Data extraction

Data were available for extraction from structured fields in CRIS. However, additional information was also provided in free-text fields. Extracting information from free-text fields was carried out using applications built using Generalised Architecture for Text Engineering (GATE) (Cunningham et al., 2013). GATE is a commonly used program featuring a variety of tools to facilitate natural language processing tasks, including information extraction from clinical notes. These applications take into account the linguistic context when extracting data from the free text, enabling automated extraction and coding of data on a large scale. They are therefore more sophisticated and validated than basic key word searches. The technique of natural language processing makes it possible to differentiate between instances where the word ‘clozapine’ is used in the context of a current prescription, or other less relevant contexts (Hayes et al., 2014).

### Main outcome measure

Readmission to SLaM services was defined as inpatient admission to SLaM services following index discharge which was within the observation period. Information about readmission was extracted from structured fields.

Care was taken to ensure that mortality was not mistaken as a lack of readmission. Routine nationwide mortality tracing linked to the electronic health record was used to determine mortality of all causes (Chang et al., 2010). All death certifications in the UK are linked to an NHS number (a unique UK NHS medical record identifier), which are all checked monthly against the national mortality database and kept up-to-date.

### Main exposure

The main exposure of interest was the antipsychotic regime established at point of discharge and sustained during transition to community settings. This was determined based on an antipsychotic prescription being given during the inpatient episode and the same drug being prescribed again at some point within the six weeks after they were discharged. Six weeks was our chosen interval to ensure all documents regarding discharge were captured, as there may sometimes be a delay between discharge and the clinician entering notes online. Information regarding medication regimens was extracted from pharmacy data, structured fields and free-text fields (with the use of natural language processing applications) in the SLaM Case Register (Hayes et al., 2014). Where patients had not been prescribed clozapine at any stage during the observation period, the first inpatient episode where the patient was prescribed an antipsychotic was selected. If patients had been prescribed clozapine, then the first discharge on clozapine was used, even if the patient had been discharged on another antipsychotic at an earlier date. The follow-up period commenced at the first discharge date where patients were prescribed clozapine or a conventional antipsychotic during the observation period, through to their first readmission, date of death, or the end of the observation period (31 December 2014), whichever occurred first.

### Covariates

We examined a number of demographic, socio-economic and clinical confounders. For this study, we derived a number of potential confounding variables from the Health of the Nation Outcome Scale (HoNOS) instrument. HoNOS was used because it is commonly used by clinicians after routine assessments in UK mental health services as a standard measure of patient wellbeing and has been well validated (Hunter et al., 2009; Orrell et al., 1999; Pirkis et al., 2005; Wing et al., 1998).

Demographic and socioeconomic factors that we examined were: age, gender, ethnicity, marital status and deprivation. Age, gender, ethnicity and marital status were derived from structured fields in CRIS. Age was calculated on the discharge date, and was converted into a categorical variable of three age categories. Ethnicity was collapsed into the following subcategories: white, other White, South Asian, East Asian, Caribbean, other Black and mixed or unknown. Marital status included whether patients were married or cohabiting. Socioeconomic status was measured using an area-level index of multiple deprivation based on the patients’ residence. This index includes several area-level domains of deprivation, provided by the 2001 national UK census (barriers to services and housing, crime, education, employment, income, health, living). Each domain was weighted according to its importance. The address of the patient used was the one recorded closest to the time they entered the study, with a separate category assigned to homeless patients.

Further clinical confounders included in the analysis were: diagnosis, HoNOS subscales of agitated behaviour, hallucinations and delusions, depressed mood and prior community treatment orders (CTOs) or depot medication. Multiple diagnoses may be given over the time that patients are in contact with SLaM services. We selected the diagnosis assigned closest to index discharge, and was categorised in accordance to ICD-10 diagnoses (F20, F25) and were extracted from free text using GATE, described above, and structured fields. The HoNOS subscales of agitated behaviour, hallucinations and delusions, and depressed mood, assessed the severity of symptoms. The HoNOS scores were extracted from structured fields and collapsed into three categories of severity from the original five due to small cell size. The first of the three categories contained the original first category: no problem. The second of the three categories contained the original second category: minor problem requiring no action. The third of the three categories contained the original third, fourth and fifth categories respectively defined as: mild problem but definitely present, moderately severe problem, and severe to very severe problem. These scores were based on the HoNOS which had been administered closest to but prior to index discharge. However, if prior scores were not available, data from the HoNOS was administered on the closest date after the index discharge.

Additional mental and physical health problems comprised HoNOS subscales of self-injury, drinking or drug taking, cognitive problems, other mental problems (phobic, anxiety, obsessive-compulsive, mental strain/tension, dissociative, somatoform, eating, sleep, sexual, other) and physical illness. Functional status comprised HoNOS subscales of daily living problems, living condition problems, occupational problems and relationship problems. As explained above, the scores were extracted from structured fields and collapsed into three categories of severity, and were assigned closest to index discharge, but preferentially beforehand.

Substance use disorders comprised diagnoses of alcohol use disorder (ICD-10: F10) or opioid use disorder (ICD-10: F11) any time prior to index discharge. This information was extracted from free text using GATE described above, and structured fields.

CTOs, legal orders under which a patient must accept treatment, and long-acting depot injections are indicated for patients non-adherent to their medication regimens, and can be administered in England and Wales under the Mental Health Act 2007. As such, a variable for whether patients had ever had CTOs or depots prior to index discharge was used in a sensitivity analysis, as a marker of potential non-adherence, based on the view that those with past non-adherence may be more likely to be non-adherent during follow-up.

### Statistical analysis

Statistical analysis was carried out using Stata, version 13. Demographic and other details pertaining to the confounding variables described above were first defined for the study cohort as a whole. Patients who were prescribed clozapine during the follow-up period were then compared to those not prescribed clozapine with respect to these potential confounding variables, using Pearson’s chi-squared test. Cox regression procedures were used, after checking the proportional hazard assumption, to model associations between clozapine and risk of readmission. An alternative analysis comparing risk of readmission between clozapine and olanzapine was also carried out. Several Cox analyses were modelled, including a crude analysis, and multivariable analyses controlling for each of the aforementioned categories of covariates, with the final model adjusting for all those examined. Propensity scores were used to address the issue of confounding-by-indication. These scores indicated the probability of being prescribed clozapine within the observation period, and included factors included in the fully adjusted model. The propensity scores were used in two ways. Firstly, a fully adjusted Cox analysis was carried out with the propensity score included as a covariate replacing the other potential confounder. Secondly, another fully adjusted Cox model included only those who had at a near-equal probability of being prescribed clozapine or not prescribed clozapine based on their propensity scores, in order to restrict the analysis to patients at a similar stage in their illness.

Next, the following fully adjusted sensitivity analyses were carried out: (a) excluding patients treated with prior depot medication or CTOs (as noncompliance and not taking medication could mean that these patients are being incorrectly assigned to the exposure group); (b) restricting the sample to patients diagnosed with ICD-10 F20 schizophrenia (to ensure the association persisted in the schizophrenia group enabling a comparison of these results with other studies which focus on schizophrenia); (c) excluding patients treated in one of the four London boroughs (Lewisham) as pharmacy data regarding clozapine exposure was incomplete in that borough; (d) comparing patients prescribed clozapine with those prescribed olanzapine at discharge; (e) excluding patients prescribed fewer than two antipsychotics prior to index discharge; (f) restricting our cohort to those who were at risk of being both treated or untreated with clozapine (based on propensity scores); (g) excluding patients outside our geographic catchment.

A Kaplan–Meier curve was produced to visualise the fully adjusted Cox regression.

## Results

**Figure 1.**
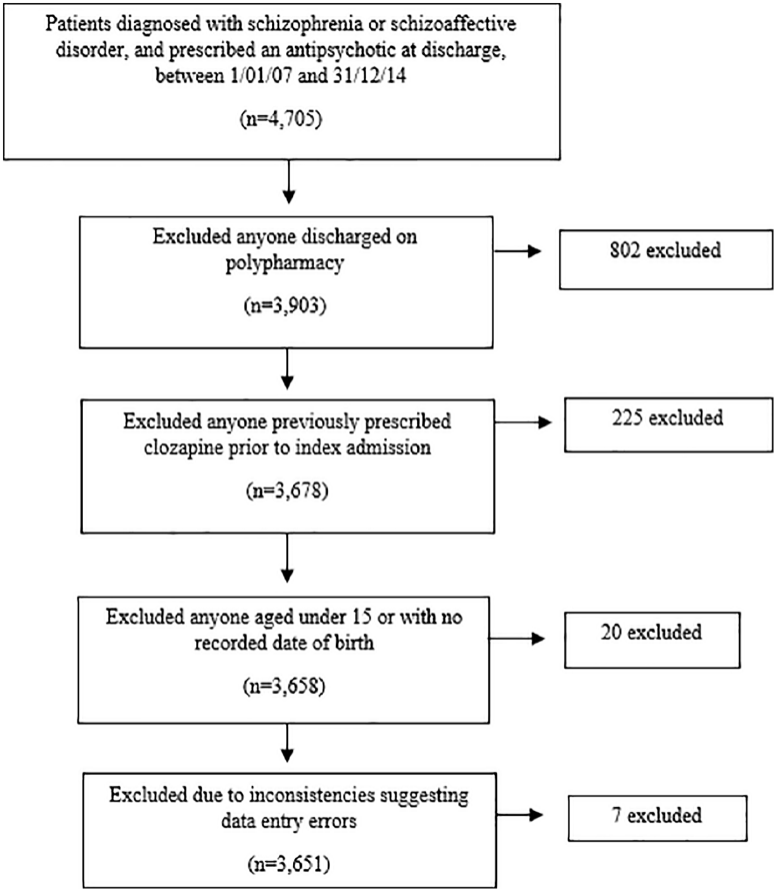
Flow chart outlining process undertaken to attain final sample size.

Over the seven-year observation period between 1 January 2007–31 December 2014, 4705 patients with a diagnosis of schizophrenia or schizoaffective disorder and sufficient data for initial inclusion were identified. After applying the inclusion criteria and data cleaning the final sample for analysis included 3651 patients, as explained by Figure 1.

The mean follow-up period was 817 days, with a standard deviation (SD) of 782 days. The total follow-up time was 8163 person-years. The GATE application that was used to extract data on antipsychotic prescriptions from electronic patient records was validated against manual review of 30 records, with a precision (positive predictive value) of 93%.

Table 1 provides data on patient characteristics for the sample used in this analysis. The ages of patients ranged from 15–95 years, with a mean age of 40.1 years and a standard deviation of 14.8. The majority of patients were male (58.6%), and reflected the ethnic diversity of South London, with nearly two-thirds of patients with a black, Asian or mixed ethnic background. Two hundred and two patients (5.5%) were prescribed clozapine and 3449 (94.5%) were prescribed another antipsychotic drug. The majority of patients were diagnosed with schizophrenia (89.3%) and experienced problems with hallucinations and delusions (71%).

**Table 1.** Sample characteristics patient cohort, including South London and Maudsley NHS Foundation Trust (SLaM) patients with schizophrenia and schizoaffective disorder, discharged on antipsychotic monotherapy between 1 January 2007–31 December 2014, and met the inclusion criteria (*n*=3651).

**Risk factors**	*n* Individuals
	(Percentage within category)
**Total**	3651 (100%)
**Taking clozapine during follow-up period**	
No	3449 (94.5%)
Yes	202 (5.5%)
**Demographic and socioeconomic factors**	
Age (mean 40.1, SD 14.8, range 15–95 years)	
15–34 years	1494 (40.9%)
35–54 years	1620 (44.4%)
55 years and over	537 (14.7%)
Gender	
Female	1512 (41.4%)
Male	2139 (58.6%)
Ethnicity	
White	978 (26.8%)
Other White	277 (7.6%)
South Asian	71 (1.9%)
East Asian	139 (3.8%)
Caribbean	484 (13.3%)
Other Black	1413 (38.7%)
Mixed unknown	289 (7.9%)
Married or cohabiting	
No	3278 (89.8%)
Yes	373 (10.2%)
Deprivation level in area of residence (tertiles)	**3585**
Low levels of deprivation	1155 (32.2%)
Medium levels of deprivation	1154 (32.2%)
High levels of deprivation	1156 (32.3%)
Homeless	120 (3.4%)
**Diagnosis and severity of symptoms**	
Diagnosis	
Schizophrenia (ICD10 code- F20)	3259 (89.3%)
Schizoaffective disorder (ICD10 code-F25)	392 (10.7%)
Agitated behaviour	**3561**
Not a problem	2083 (58.5%)
Minor problems only	776 (21.8%)
Significant problem	702 (19.7%)
Hallucinations and delusions	**3552**
Not a problem	1029 (29.0%)
Minor problems only	814 (22.9%)
Significant problem	1709 (48.1%)
Depressed mood	**3555**
Not a problem	1809 (50.9%)
Minor problems only	1043 (29.3%)
Significant problem	703 (19.8%)
Ever had a community treatment order	
No	3581 (98.1%)
Yes	70 (1.9%)
Ever been on depot medication	
No	3266 (89.5%)
Yes	385 (10.6%)
**Additional mental and physical health problems**	
Self-injury	**3559**
Not a problem	3166 (89.0%)
Minor problems only	189 (5.3%)
Significant problem	204 (5.7%)
Problem-drinking or drug taking	**3528**
Not a problem	2566 (72.7%)
Minor problems only	336 (9.5%)
Significant problem	626 (17.7%)
Cognitive problems	**3554**
Not a problem	2217 (62.4%)
Minor problems only	761 (21.4%)
Significant problem	576 (16.2%)
Other mental problems	**3551**
Not a problem	1335 (37.6%)
Minor problems only	910 (25.6%)
Significant problem	1306 (36.8%)
Physical illness	**3550**
Not a problem	2470 (69.6%)
Minor problems only	512 (14.4%)
Significant problem	568 (16.0%)
**Functional status**	
Daily living problems	**3527**
Not a problem	1786 (50.6%)
Minor problems only	891 (25.3%)
Significant problem	850 (24.1%)
Living conditions problems	**3457**
Not a problem	1885 (54.5%)
Minor problems only	733 (21.2%)
Significant problem	839 (24.3%)
Occupational problems	**3449**
Not a problem	1349 (39.1%)
Minor problems only	1008 (29.2%)
Significant problem	1092 (31.7%)
Relationship problems	**3523**
Not a problem	1390 (39.5%)
Minor problems only	1020 (29.0%)
Significant problem	1113 (31.6%)
**Substance use disorders**	
Ever diagnosed with alcohol use disorder	
No	3355 (91.9%)
Yes	296 (8.1%)
Ever diagnosed with opioid use disorder	
No	3591 (98.4%)
Yes	60 (1.6%)

ICD-10: International Classification of Diseases 10; SD: standard deviation.

**Figure 2.**
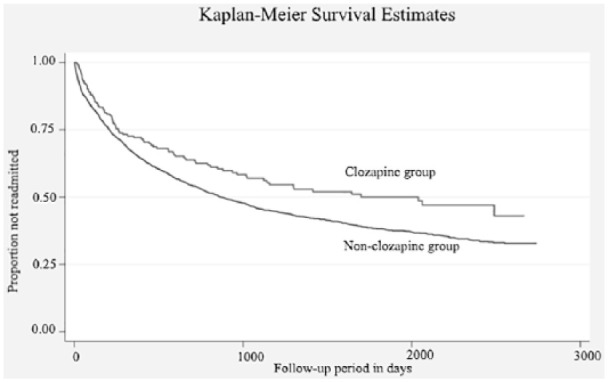
Kaplan–Meier Curves comparing those who were newly prescribed clozapine with those who were not: displaying the proportion of patients with schizophrenia and schizoaffective disorder who were not readmitted into inpatient mental health services over the follow-up period (*n*=3651).

Table 2 shows comparisons between patients with or without exposure to clozapine. Those prescribed clozapine were more likely to be male, single and to have received schizophrenia as a primary diagnosis, to a statistically significant level (*p*<0.05). Patients prescribed clozapine were more likely to function more poorly with regards to problems with daily living and occupation, and have more severe psychopathology, including problems with agitated behaviour, hallucinations and delusions, depressed mood, drinking or drug taking, cognitive problems and other mental problems. They were also more likely to have been on prior depot medication or CTOs. A smaller percentage of patients newly prescribed clozapine were readmitted into mental health services (44.1% (*n*=89) compared with of non-clozapine users 54.2% (*n=*1869) (*p*<0.05)). Figure 2 displays Kaplan–Meier curves for patients prescribed clozapine and not prescribed clozapine.

**Table 2.** Sample characteristics comparing those prescribed clozapine with those not prescribed clozapine, including South London and Maudsley NHS Foundation Trust (SLaM) patients with schizophrenia and schizoaffective disorder, discharged on antipsychotic monotherapy between 1 January 2007–31 December 2014, and met the inclusion criteria (*n*=3651).

Risk factors	Not prescribed clozapine	Prescribed clozapine
	*n (%)*	*n (%)*
**Total**	3449	202
**Readmission** ^a^	1869 (54.2%)	89 (44.1%)
**Demographic and socioeconomic factors**		
Age		
15–34 years	1401 (40.6%)	93 (46.0%)
35–54 years	1533 (44.5%)	87 (43.1%)
55 years and over	515 (14.9%)	22 (10.9%)
Gender^a^		
Female	1446 (41.9%)	66 (32.7%)
Male	2003 (58.1%)	136 (67.3%)
Ethnicity^a^		
White	906 (26.3%)	72 (35.6%)
Other White	263 (7.6%)	14 (6.9%)
South Asian	63 (1.8%)	8 (4.0%)
East Asian	133 (3.9%)	6 (3.0%)
Caribbean	472 (13.7%)	12 (5.9%)
Other Black	1338 (38.8%)	75 (37.1%)
Mixed or unknown	274 (7.9%)	15 (7.4%)
Married or cohabiting		
No	3089 (89.6%)	189 (93.6%)
Yes	360 (10.4%)	13 (6.4%)
Deprivation level in area of residence (tertiles)		
Low levels of deprivation	1078 (31.8%)	77 (38.9%)
Medium levels of deprivation	1097 (32.4%)	57 (28.8%)
High levels of deprivation	1098 (32.4%)	58 (29.3%)
Homeless	114 (3.4%)	6 (3.0%)
**Diagnosis and severity of symptoms**		
Diagnosis		
Schizophrenia (ICD10 code- F20)^a^	3075 (89.2%)	184 (91.1%)
Schizoaffective disorder (ICD10 code- F25)	374 (10.8%)	18 (8.9%)
Agitated behaviour^a^		
Not a problem	1939 (57.7%)	144 (72.4%)
Minor problems only	749 (22.3%)	27 (13.6%)
Significant problem	674 (20.1%)	28 (14.1%)
Hallucinations and delusions		
Not a problem	980 (29.2%)	49 (24.8%)
Minor problems only	775 (23.1%)	39 (19.7%)
Significant problem^a^	1599 (47.7%)	110 (55.6%)
Depressed mood		
Not a problem	1702 (50.8%)	107 (53.8%)
Minor problems only	979 (29.2%)	64 (32.2%)
Significant problem	675 (20.1%)	28 (14.1%)
Ever had a community treatment order^a^		
No	3390 (98.3%)	191 (94.5%)
Yes	59 (1.7%)	11 (5.5%)
Ever been on depot medication^a^		
No	3097 (89.8%)	169 (83.7%)
Yes	352 (10.2%)	33 (16.3%)
**Additional mental and physical health problems**		
Self-injury		
Not a problem	2987 (88.9%)	179 (90.4%)
Minor problems only	176 (5.2%)	13 (6.6%)
Significant problem	198 (5.9%)	6 (3.0%)
Problem-drinking or drug taking^a^		
Not a problem	2413 (72.4%)	153 (77.7%)
Minor problems only	314 (9.4%)	22 (11.2%)
Significant problem	604 (18.1%)	22 (11.2%)
Cognitive problems^a^		
Not a problem	2114 (63.0%)	103 (52.0%)
Minor problems only	708 (21.1%)	53 (26.8%)
Significant problem	534 (15.9%)	42 (21.2%)
Other mental problems		
Not a problem	1265 (37.7%)	70 (35.4%)
Minor problems only^a^	840 (25.1%)	70 (35.4%)
Significant problem	1248 (37.2%)	58 (29.3%)
Physical illness		
Not a problem	2343 (69.9%)	127 (64.1%)
Minor problems only	474 (14.1%)	38 (19.2%)
Significant problem	535 (16.0%)	33 (16.7%)
**Functional status**		
Daily living problems^a^		
Not a problem	1709 (51.4%)	77 (38.7%)
Minor problems only	831 (25.0%)	60 (30.2%)
Significant problem	788 (23.7%)	62 (31.2%)
Living conditions problems		
Not a problem	1767 (54.2%)	118 (60.8%)
Minor problems only	697 (21.4%)	36 (18.6%)
Significant problem	799 (24.5%)	40 (21.0%)
Occupational problems		
Not a problem	1285 (39.5%)	64 (33.3%)
Minor problems only	943 (29.0%)	65 (33.9%)
Significant problem	1029 (31.6%)	65 (32.8%)
Relationship problems		
Not a problem	1318 (39.6%)	72 (36.5%)
Minor problems only	956 (28.7%)	64 (32.5%)
Significant problem	1052 (31.6%)	61 (31.0%)
**Substance use disorders**		
Ever diagnosed with alcohol use disorder		
No	3167 (91.8%)	188 (93.1%)
Yes	282 (8.2%)	14 (6.9%)
Ever diagnosed with opioid use disorder		
No	3391 (98.3%)	200 (99.0%)
Yes	58 (1.7%)	2 (1.0%)

ICD-10: International Classification of Diseases 10.

a Value of *p*<0.05 for comparison between those who were and were not prescribed clozapine.

Table 3 displays Cox regression models investigating the association between clozapine exposure and readmission. Clozapine was associated with a reduced risk of readmission in the crude analysis, hazard ratio (HR) 0.73 (95% confidence interval (CI): 0.59–0.91; *p*=0.004), and fully adjusted models, adjusted hazard ratio (aHR) 0.79 (95% CI: 0.64–0.99; *p*=0.043). This association also persisted when carrying out fully adjusted sensitivity analyses: excluding noncompliance (aHR 0.73 (95% CI: 0.57–0.94); *p*=0.013), restricting to schizophrenia (aHR 0.76 (95% CI: 0.60–0.96); *p*=0.024), excluding patients from Lewisham (aHR 0.74 (0.56–0.96); *p*=0.025). This association also persisted in a fully adjusted model based on propensity scores, which included only those who had at a near-equal probability of being prescribed clozapine or not prescribed clozapine, aHR 0.80 (95% CI: 0.64–1.00; *p*=0.047). Clozapine also reduced risk of readmission more than olanzapine in a fully adjusted model, aHR 0.76 (95% CI: 0.60–0.96; *p*=0.021). A further sensitivity analysis was carried out to fully exclude all patients outside the designated geographic catchment, as external patients are more likely to have been those treated by the National Psychosis Unit, Psychiatric Intensive Care Units, and forensic units, which tend to admit more patients from outside the boroughs due to their clinical complexity. These patients are therefore less likely to have readmission data. In this sensitivity analysis, this association was not present, HR 0.87 (95% CI: 0.69–1.09; *p*=0.228). A final sensitivity analysis was also carried out to exclude all patients who had been prescribed fewer than two antipsychotics, where this association was also no longer significant, HR 0.79 (95% CI: 0.62–1.00; *p*=0.054).

**Table 3.** Multivariable Cox regression analyses for the associations between newly prescribed clozapine and risks of readmission in patients with schizophrenia and schizoaffective disorder (*n*=3651).

Prescribed clozapine during follow-up period	Hazard ratio (95% CI)	*p* Values
Crude	0.73 (0.59–0.91)	0.004
Crude clozapine versus olanzapine	0.68 (0.54-0.84)	0.001
Adjusted for age and gender	0.73 (0.59–0.90)	0.003
Adjusted for socioeconomic and demographic factors^a^	0.73 (0.59–0.91)	0.005
Adjusted for diagnosis and severity of symptoms^b^	0.78 (0.63–0.97)	0.027
Adjusted for additional mental and physical factors^c^	0.75 (0.61–0.93)	0.009
Adjusted for functional status^d^	0.75 (0.60–0.94)	0.011
Adjusted for substance use disorders^e^	0.74 (0.59–0.91)	0.005
Fully adjusted^f^	0.79 (0.64–0.99)	0.043
Adjusted by using propensity score as a confounding variable	0.78 (0.63–0.98)	0.032
Sensitivity analyses		
Fully adjusted^f^ excluding potentially noncompliant individuals (those who had received depot medication or had been placed on a community treatment order at any time in SLaM services)	0.73 (0.57–0.94)	0.013
Fully adjusted^f^ including only those diagnosed with schizophrenia	0.76 (0.60–0.96)	0.024
Fully adjusted^f^ excluding those treated in Lewisham	0.74 (0.56–0.96)	0.025
Fully adjusted^f^ comparing with those who were prescribed olanzapine at discharge	0.76 (0.60–0.96)	0.021
Fully adjusted excluding people with fewer than two antipsychotics prescribed prior to index discharge	0.79 (0.62–1.00)	0.054
Fully adjusted^f^ including only those who had at a near-equal probability of being prescribed clozapine or not prescribed clozapine (based on propensity scores)	0.80 (0.64–1.00)	0.047
Fully adjusted^f^ excluding patients outside geographic catchment	0.87 (0.69–1.09)	0.228

CI: confidence interval.

Reference group consists of patients taking antipsychotics apart from clozapine, unless specified.

a Age, gender, ethnicity, marital status, deprivation level in area of residence.

b Diagnosis, aggressive behaviour, hallucinations and delusions, depressed mood.

c Non-accidental self-injury, physical illness.

d Problems in activities of daily living, living conditions, occupation, social relationships.

e Ever having had alcohol or opioid use disorder diagnoses.

f All of the above.

## Discussion

This study tested the hypothesis that newly prescribed clozapine use, in the treatment of schizophrenia and schizoaffective disorder, is associated with a decreased risk of readmission compared with treatment with conventional antipsychotics. We found evidence that clozapine was associated with a moderately lower risk of readmission, supporting the hypothesis. Although patients who were newly prescribed clozapine were more ill at baseline, had poorer functional status and more severe psychopathology, they had a lower risk of readmission as compared with patients not prescribed clozapine. This association remained after adjusting for a large variety of confounding factors including: demographic, socioeconomic and clinical factors. This association persisted following several sensitivity analyses and the use of propensity scores to address confounding-by-indication. However, when carrying out the sensitivity analysis excluding all patients outside the designated geographic catchment, and the sensitivity analysis excluding patients who had been prescribed fewer than two antipsychotics prior to index discharge, the direction of the association remained and the effect estimate was similar, but the association was no longer significant. This is most likely due to the substantial reduction in sample size and therefore loss of power for these analyses.

These findings are consistent with several other studies, which have also found an association between clozapine and a decreased risk of readmission. For example, a retrospective analysis of 117 schizophrenia patients prescribed clozapine in South Korea found that their readmission rates were lower after initiation on clozapine compared with beforehand, and that this association was still present five years after initiation on clozapine (*p*<0.01) (Ahn et al., 2005). This study was restricted to patients with schizophrenia. When we restricted our sample to only patents with schizophrenia we still saw a significant protective effect of clozapine. These findings are also consistent with more recent studies. A recent definitive meta-analysis found that clozapine was associated with a reduction in the proportion of admissions into hospital compared with other antipsychotics including olanzapine (risk ratio = 0.74; 95% CI: 0.69–0.80; *p*<0.001) (Land et al., 2017). A recent cohort study using nationwide databases included traditional Cox proportional hazards models and within-individual Cox proportional hazards models. Clozapine was also associated with a reduced risk of readmission (HR 0.53; 95% CI: 0.48–0.58) (Tiihonen et al., 2017). However, existing research has failed to adjust for important time-dependent potential confounders such as changes in symptoms. As previously discussed, this highlights ongoing limitations in current research with regards to limited controlling for confounding variables.

This study had several strengths. Firstly, we were able to capture and adjust for a number of important clinical, sociodemographic and socio-economic variables, which limited the possibility of residual confounding. Confounding-by-indication was also addressed using propensity scores and a sensitivity analysis excluding patients who had been prescribed fewer than two antipsychotics prior to index discharge. Although this study did not directly address the possibility that the promising results associated with clozapine may be due to more frequent specialist follow-up in patients taking clozapine compared with those taking other antipsychotics, as is common clinical practice in the UK, this has been addressed in a previous study, which found that this is not the case (Hayes et al., 2014). The risk of readmission for those newly prescribed clozapine was also compared with olanzapine, showing that it offers more protective effects than a commonly used antipsychotic. This also allowed comparisons with other mental health services where the distribution of antipsychotics in the non-clozapine comparison group may be different than SLaM. Also, if the non-clozapine antipsychotics prescribed within SLaM were comparatively ineffective to those prescribed within other mental health services, this may have caused an overestimation of the protective effect of clozapine. Thus, comparing clozapine with olanzapine addressed this. Moreover, as the patient sample constituted all patients with schizophrenia and schizoaffective disorder in contact with SLaM mental health secondary care which serves a defined geographic catchment over a seven-year period, it is likely that these data are representative of patients with schizophrenia and schizoaffective disorder living in suburban and urban areas. This is because SLaM is a near-monopoly provider of mental health services within its geographic catchment.

This study also had a number of limitations. Firstly, as the focus of this investigation was the effect of being discharged on newly prescribed clozapine on hospital readmission, it did not elucidate the long-term effect of being prescribed clozapine. This study limited the cohort to those newly prescribed clozapine in order to make clozapine and non-clozapine groups more comparable. A further limitation is that as we measured the first readmission after discharge and not subsequent discharges, changes in clinical course were not measured, so the longevity and patterns of the protective effects of clozapine on readmission are uncertain. Moreover, since only patients on monotherapy were included in this investigation, these results may not be applicable to patients on polypharmacy. In addition, residual confounding may still be present due to factors which were not adjusted for in this investigation. This includes factors not mentioned by clinicians, as extracted information about patients relied on clinical records, in addition to lifestyle factors, such as the possibility that patients on clozapine may be exposed to less stress following discharge if, for example, they are not expected to work and contribute socially. In particular, confounding-by-indication is an important issue in observational studies investing the impact of medications. For example, since clozapine is a third-line antipsychotic, patients who are newly prescribed clozapine are more likely to be at a later stage in the course of their treatment and illness than those who have been newly prescribed another antipsychotic. Although propensity scores were used to address this as well as a sensitivity analysis excluding patients who had been prescribed fewer than two antipsychotics prior to index discharge, confounding-by-indication cannot be completely ruled out and there is still potential for bias. However, as previously discussed, this difference would most likely lead to the comparison group being healthier at baseline (as borne out by our finding that those on clozapine had worse psychopathology as baseline) and less likely to be readmitted. Consequently, any such bias would have mostly likely produced an underestimate of the protective effect of clozapine so the protective effect of clozapine might be expected to be at least as strong as reported here. Indeed, some variables that we adjusted for may have been suboptimally measured due to limitations in data collection. For example, patients likely to have been non-compliant with their medication, were defined as those who had ever been treated by a depot or subject to a CTO. At 12% of our cohort, this is likely to be an underestimate of the prevalence of non-compliance, as it is difficult to identify patients who do not take their prescribed regular medication, and thus include such patients in our analyses. Clozapine is well recognised for its effectiveness in treating treatment-resistant schizophrenia (Wimberley et al., 2017). It is likely the mechanism for clozapine reducing the risk of readmission is that it improves certain domains of mental health which are normally problematic for patients with schizophrenia and schizoaffective disorder, and warrant readmission. However, this investigation did not compare the severity of these domains before and after initiation on clozapine. This was because HoNOS scores are not always recorded frequently enough in clinical practice to measure these changes over small time periods. Indeed, the exact causal mechanism that leads to the benefits of clozapine remain unclear. It is important that subsequent studies further elucidates this, and the influence concomitant treatments such as psychotherapy may have on clinical outcomes, as patients may undergo both pharmacological and psychological therapies. Future research should take the aforementioned limitations into account, by investigating the effects of long-term prescribing of clozapine, including patients on polypharmacy, and adjusting for potential confounding variables not considered by this investigation. Lastly, power appears to have been an issue in this study, as shown by the sensitivity analysis excluding all patients from the geographic catchment, including those who were homeless. This is likely to be a power issue as this sensitivity analysis excluded 171 patients, with only a slight change in the HR but a large change in the *p*-value.

The results of this study have important clinical implications, which may be relevant for future clinical practice. Clozapine appears to reduce readmission rates into secondary mental health services. This highlights a potential benefit of prescribing clozapine for use in treatment-resistant schizophrenia and schizoaffective disorder, which is currently underused in clinical practice, despite treatment guidelines (Howes et al., 2012). However, more research addressing the above limitations is required, before such benefit may be translated into clinical practice.
